# Predicting structured metadata from unstructured metadata

**DOI:** 10.1093/database/baw080

**Published:** 2016-05-17

**Authors:** Lisa Posch, Maryam Panahiazar, Michel Dumontier, Olivier Gevaert

**Affiliations:** ^1^GESIS – Leibniz Institute for the Social Sciences, Cologne, Germany; ^2^Institute for Web Science and Technologies, University of Koblenz-Landau, Koblenz, Germany; ^3^Stanford Center for Biomedical Informatics Research, Department of Medicine, Stanford University, Stanford, CA, USA

## Abstract

Enormous amounts of biomedical data have been and are being produced by investigators all over the world. However, one crucial and limiting factor in data reuse is accurate, structured and complete description of the data or data about the data—defined as metadata. We propose a framework to predict structured metadata terms from unstructured metadata for improving quality and quantity of metadata, using the Gene Expression Omnibus (GEO) microarray database. Our framework consists of classifiers trained using term frequency-inverse document frequency (TF-IDF) features and a second approach based on topics modeled using a Latent Dirichlet Allocation model (LDA) to reduce the dimensionality of the unstructured data. Our results on the GEO database show that structured metadata terms can be the most accurately predicted using the TF-IDF approach followed by LDA both outperforming the majority vote baseline. While some accuracy is lost by the dimensionality reduction of LDA, the difference is small for elements with few possible values, and there is a large improvement over the majority classifier baseline. Overall this is a promising approach for metadata prediction that is likely to be applicable to other datasets and has implications for researchers interested in biomedical metadata curation and metadata prediction.

**Database URL:** http://www.yeastgenome.org/

## Introduction

Enormous amounts of biomedical data have been and are being produced by investigators all over the world. This is mainly due to advancements in molecular technologies that have enabled extensive profiling of biological samples and have unleashed a myriad of omics data such as gene expression, microRNA expression, DNA methylation and DNA mutation data. However, during the last decade, journals, investigators, funding agencies, etc. realized that this data should be stored and shared with other investigators. Several databases were created in the process to house this data and make it available to the community at large, such as the NCBI databases for microarray data; Gene Expression Omnibus (GEO) (1) and sequence data; the database of Genotypes and Phenotypes (dbGAP) (2). This data repurposing—the use of data beyond what the original investigators envisioned—has been very fruitful for the biomedical and biological community by allowing meta-analysis studies to discover novel biology hidden in each single dataset.

In this article, we predict the structured metadata from textual metadata elements. With predicting metadata, we aim to improve the quality and quantity of metadata. This improvement for biomedical datasets is crucial to drive the next paradigm shift in data reuse. Tackling the metadata problem will remove hurdles in mapping datasets to each other and time consuming efforts to find datasets for an investigator's disease of interest. It will allow streamlined meta-analysis due to easier discovery of similar datasets owing to harmonized metadata annotations.

Despite the development of community standards to describe an experiment (e.g. Minimum Information About a Microarray Experiment; MIAME) (3) or a dataset (e.g. Health Care and Life Sciences; HCLS dataset description) (4), these are often poorly implemented, making it difficult to find, access and reuse data. However, one crucial and limiting factor in data reuse is accurate, structured and complete description of the data or data about the data—defined as metadata. Overall, current metadata is often of low quality, and many fields are either altogether absent, erroneous or inconsistent. For example, the largest database of gene expression studies, the GEO microarray database, contains over 50 000 studies, more than 1.3 million samples, and is still growing. However, only 15 metadata fields are consistently annotated out of 32 fields. Thousands of records have empty values. The majority of the samples have fewer than four sample level annotations and they suffer from a spectacular lack of consistency (e.g. there are over 30 different ways to specify the ‘age in years’ in the GEO database). It takes time and effort to create well-specified metadata, and investigators view the task of metadata authoring (or data annotation) to be a burden that may benefit other scientists, but not the team that did the work in the first place. There is minimal verification for the correctness of metadata during submission. Because of this, valuable information may not be reusable by other researchers.

Our solution is to leverage textual descriptions for generating structured data based on context and corpus statistics. The main contribution of this paper is to explore whether unstructured gene expression sample metadata contains information, which can be exploited for predicting structured metadata using traditional text mining methods based on term frequency-inverse document frequency (TF-IDF). Furthermore, we will explore whether topic models are able to identify semantically meaningful topics in the unstructured text contained in sample metadata, and whether this topic representation is suitable for training supervised classifiers, with the aim of predicting the values of structured metadata elements compared to the TF-IDF approach.

Topic models are a widely used method for the analysis of textual corpora and other discrete data. We use Latent Dirichlet Allocation (5), one of the most popular topic models, for discovering the latent topics which are present in the metadata of gene expression samples. Topic models provide the opportunity to reduce the description length of the documents as compared to TF-IDF, and they also produce semantically meaningful features in the form of latent topics (5). In the experiments presented in this article, these topics are used as features for training supervised classifiers, with the aim of predicting metadata values. We compare the classification results of topic models to those produced by classifiers that use TF-IDF features to investigate how much discriminatory information is lost by this reduction of document description length.

This article is structured as follows. The ‘Dataset’ section describes the characteristics of the dataset that we used for our experiments, and ‘Methods’ section describes the details of our experimental setup. The results of our experiments are reported in the ‘Results’ section. The ‘Discussion’ section 5 discusses the results and provides directions for future work. The ‘Conclusion’ section concludes this article.

## Dataset

For all our experiments, we used data from GEO, a database of gene expression data which contains experimental metadata largely authored by original data submitters. The GEO database contains over 1.3 million records. It contains over 50 000 studies, called ‘series’ in GEO. A series (identified with GSE00001 in GEO) organizes samples into a data set defining an experiment. Each study contains 30 samples on average. A sample (identified with GSE00001 in GEO) describes the set of molecules that are being probed and references a single platform used to generate its molecular abundance data (GEO) (1). Note that in some cases samples can belong to multiple series which complicates the evaluation approach (see below). In this article, we used data at the sample level for all studies. Each study is annotated with up to 32 metadata fields representing the conditions under which the sample was handled, the manipulations it underwent and the measurements of each element derived from it. These metadata fields can be either structured or unstructured.

**Figure 1.**
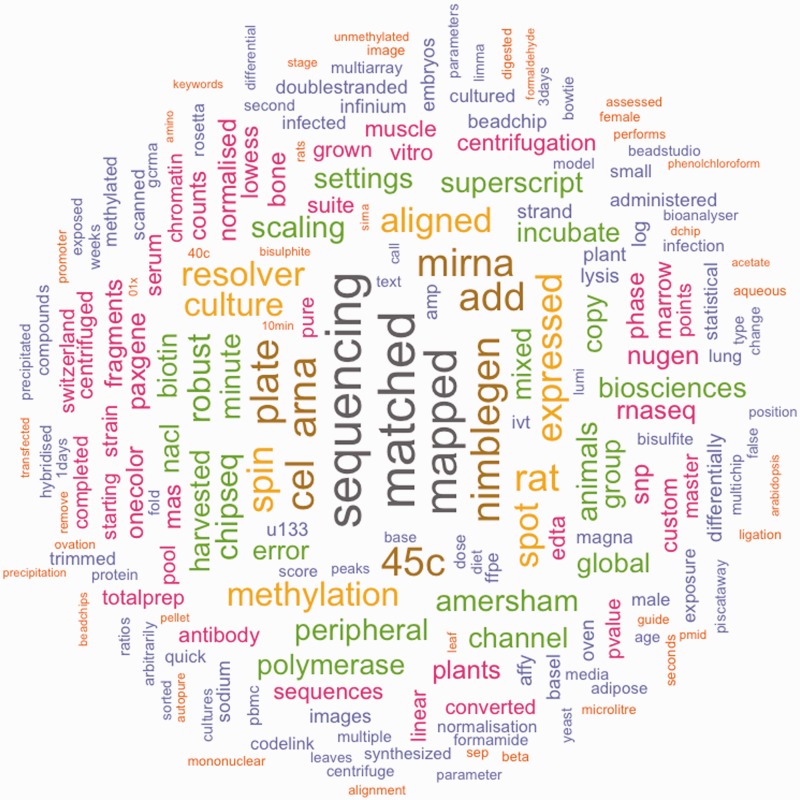
Frequent terms in the unstructured text values of GEO metadata values. This figure shows terms which are frequently used in the unstructured elements of the GEO dataset. Colors and size correspond to the frequency of a term.

We define a structured element as a metadata element which contains a single concept, such as the organism from which the material was derived. Values of structured elements have the potential to be mapped to concepts contained in a controlled vocabulary. Analogously, we define an *unstructured element* as a metadata element which contains any other form of textual description provided by the user. The value domain of unstructured elements is free text and cannot be predicted in any meaningful way. Tables 1 and 2 show a list of the structured and unstructured metadata fields, respectively. Next, we define *document* as the combined unstructured text of one metadata record. Figure 1 shows which terms occur most frequently in these documents.

**Table 1.** Structured metadata fields in GEO.

Field	Description
GPL	The unique GEO accession number for the platform (GSE00001). A platform is, essentially, a list of probes that define what set of molecules may be detected, e.g. GPL13653 for the Affymetrix GeneChip Rat Genome U34A Array.
Type	Type of the sample e.g., RNA or DNA.
Organism	The organism(s) from which the biological material was derived, e.g. homo sapiens, drosophila melanogaster.
Molecule	Type of molecule that was extracted from the biological material, e.g. total RNA, cytoplasmic RNA.
Label	The compound used to label the extract, e.g. biotin, Cy3.

This table lists the structured metadata fields along with a description of each element.

**Table 2.** Unstructured metadata fields in GEO.

Field	Description
Title	A unique title that describes the samples.
Source Name	The biological material and the experimental variable(s), e.g., vastus lateralis muscle, exercised, 60 min.
Treatment Protocol	Treatments applied to the biological material prior to extract preparation.
Extraction Protocol	The protocol used to isolate the extract material.
Label Protocol	The protocol used to label the extract.
Hybridization Protocol	The protocols used for hybridization, blocking and washing, and any post-processing steps such as staining.
Description	Any additional information not provided in the other fields.
Data Processing	Details of how data in the matrix table were generated and calculate.

This table lists the unstructured metadata fields along with a description of each element.

## Methods

**Figure 2.**
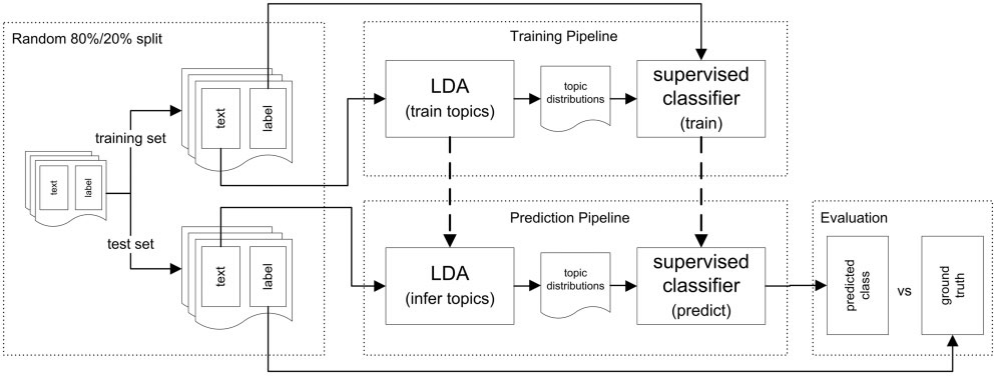
Overview of the experimental setup. This figure shows the four main components of the experimental setup: pre-processing and splitting the data into a training set and a test set, training the LDA model as well as the supervised classifier, inferring the per-document topic distributions and predicting the classes, and evaluation of the predictions. The classification setup for the classifiers using TF-IDF features is analogous: Instead of latent topics, TF-IDF values are used for representing the documents.

In this section, we describe the experimental setup and the methods used for our classification experiments. Our goal is to predict structured metadata values using a classifier trained on the latent topic distributions derived from the unstructured elements and to analyse how well this method performs compared to using the full vocabulary for document description. To accomplish this, we use the documents’ latent topic distributions inferred by LDA, as well as TF-IDF values, as features to represent the documents. For our metadata prediction experiments, we constructed a pipeline (Figure 2) for training and evaluating different supervised classifiers. The experimental setup consists of four main components for training the classifiers and evaluating their predictive performance: (i) pre-processing and splitting the data into a training set and a test set, (ii) training the LDA model, calculating TF-IDF values and training the supervised classifiers, (iii) inferring the per-document topic distributions, calculating TF-IDF values for the test set based on the document frequencies of the training set and predicting the classes and (iv) evaluation of the predictions. Figure 2 shows an overview of this setup, based on LDA features; the setup which uses TF-IDF features is analogous. The experimental setup ensures that no information from the training set is used in evaluation. For evaluation, we compare the performance of the supervised classifiers trained on the topic distributions obtained by LDA with the classifiers trained on TF-IDF features and with the performance of a majority classifier. In the remainder of this section, the individual components of the setup are described in more detail.

### Pre-processing

Gene Expression Omnibus (GEO) consists of 1 368 682 individual sample records. As a first step, we split the records randomly into a training set consisting of 80% of the records and a test set consisting of the remaining 20%. As previously explained samples belong to a series and a subset of samples can belong to multiple series. To avoid information leakage from the training to the test set, we first identified the *superseries* that each sample belongs to defined as the grouping of all series that share at least one sample. Then a superseries is either assigned to the train or test set and thus never split up. After the split, both the unstructured text and the values of the structured elements are pre-processed in preparation for the classification experiments.

#### *Unstructured* *text*

We applied standard pre-processing methods to the combined values (i.e. unstructured text) of all unstructured elements. After removing English stopwords (https://code.google.com/p/stop-words/) as well as rare and frequent words, the vocabulary consisted of 70 802 distinct terms. Specifically, words which had less than three characters and words which occurred in < 20 documents of the training set were removed, as well as the most frequently occurring 200 words of the training set. Furthermore, documents with less than five words were removed from the dataset. We did not apply stemming as unstemmed words result in more easily interpretable topics and allow the topic model to capture more subtle latent semantics.

#### Labels

**Figure 3.**
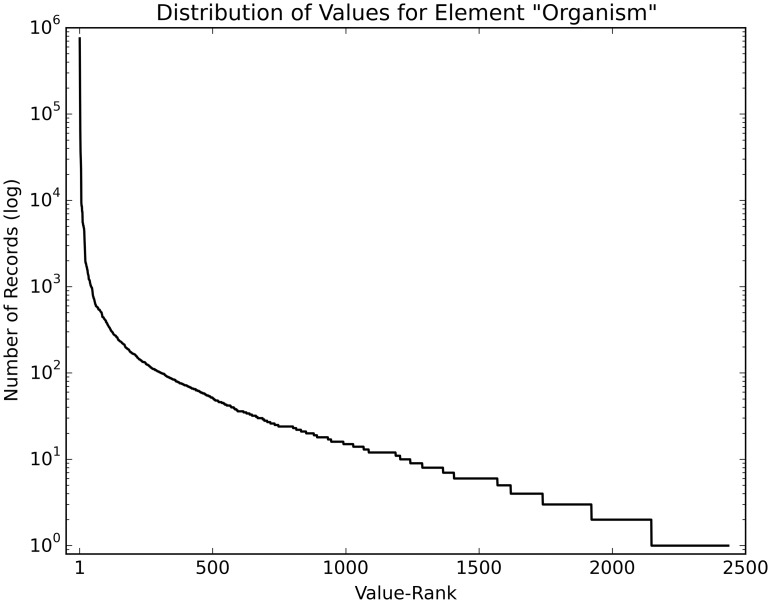
Value distribution of the element ‘Organism’. This figure shows the skewed distribution of values of the element ‘Organism’ (log scale), with the most frequently used value (homo sapiens) occurring 603 446 times and 1233 values occurring <10 times.

The structured elements of the dataset have a heavily skewed value distribution. As an example, Figure 3 shows the value distribution of the element ‘Organism’ in the training set, after minimal pre-processing (lowercasing and removing special characters). Many values are rare; 1233 values occur <10 times.

As supervised classifiers rely on being provided with a sufficiently large set of training examples for each class, we chose to exclude infrequently used element values. Specifically, we excluded values which occurred in less than 0.1% of the dataset records (i.e. values which occurred <1368 times in the dataset). Table 3 shows the number of classes which remained after excluding rare classes, for each of the structured elements.

**Table 3.** Number of classes which constitute at least 0.1% of the dataset.

Element Name	Number of classes	Example Values
GPL	123	gpl570, gpl1261, gpl96, gpl10558
Label	23	biotin, cy3, cy5_and_cy3, alexa_fluor_647
Molecule	5	total_rna, genomic_dna, polya_rna, protein
Organism	33	homo_sapiens, zea_mays, gallus_gallus
Type	7	rna, genomic, sra, protein

This table shows the number of classes which constitute more than 0.1% of the dataset, as well as example values, for each structured element.

### Model training

The training pipeline first trains a topic model on the unstructured text from the training set and subsequently trains a supervised classifier on the features resulting from the topic model. For comparison, the same type of classifier is trained on TF-IDF features.

#### Topic model

The first step of the training pipeline trains a topic model on the unstructured text of the training set. The topic model which we use for our experiments is Latent Dirichlet Allocation (LDA). This is a generative Bayesian model which was introduced by Blei et al. (5). LDA requires the number of latent topics to be set in advance, as well as the specification of two hyper-parameters: α for smoothing the document-topic distribution and β for smoothing the topic-term distribution. We built an LDA model with 150 topics on the unstructured text of the training set. This number of topics was chosen based on a document description length which is longer than the number of different values in the most diverse structured element (GPL). The hyper-parameters α and β were set to 0.1 and 0.01, respectively. These relatively low values indicate our prior beliefs that a sample consists of a mixture of relatively few topics, and that the topics in this dataset contain a mixture of a few, specific words (rather than many words of equal importance). A total of 200 iterations of Collapsed Variational Bayes inference (6) were used to train the model. In the model, each document is represented by a distribution over all 150 topics, and each topic is represented by a distribution over all terms in the vocabulary. This means that the dimensionality of the feature space, compared to representation methods such as term frequency or TF-IDF, was reduced by almost 98% (from 70 802 to 150).

#### TF-IDF

With the document frequencies of the training set, TF-IDF values are calculated for all documents in the training set.

#### *Supervised* *classifiers*

The document-topic distributions resulting from the LDA model, as well as the TF-IDF values, were used as features for representing the documents in the supervised classification part of our experiments. For classification, we used a support vector machine (SVM) with linear kernel for each of the structured elements (7). Additionally, we used a majority classifier as a baseline. The classifiers were trained on the records from the training set. The linear SVM was trained using the default value for the penalty parameter C = 1.0. All classifiers were trained and tested using the Python library scikit-learn (http://scikit-learn.org/stable/). The LDA model was built using the Stanford Topic Modeling Toolbox (http://nlp.stanford.edu/software/tmt/tmt-0.4/).

### Prediction pipeline

The prediction pipeline first uses the trained topic model to infer the document-topic distributions of the records contained in the test set, and then uses these inferred distributions for predicting the classes with the supervised classifier. To analyse how much information was lost during the dimensionality reduction, TF-IDF features are also used for prediction.

#### *Topic* *model*

For inferring the topic distribution of new, unseen documents, the topic-term distribution learned by the topic model from the training data was used. It remains unchanged as the document-topic distribution for the new documents is inferred.

#### TF-IDF

With the document frequencies from the training set, TF-IDF values are calculated for all documents in the test set.

#### *Supervised* *classifiers*

The inferred document-topic distributions, as well as the TF-IDF values, were used as a representation of the documents contained in the test set, for the prediction performed by classifiers which were trained as described above. The trained supervised classifiers were then used to predict the classes of the records from the test set.

### Evaluation

For evaluating the classifiers, we compare the performance of the different classification models by using the standard evaluation measures precision, recall and F1-Score, defined as the harmonic mean of precision and recall. The metrics we report are class averages, weighted by the support of the class (i.e. the number of occurrences of this class in the test set). Each prediction is compared to the actual class value of our ground truth (i.e. the original class of the respective sample). Additionally, we calculated the metrics for a baseline classifier in order to obtain a fair evaluation of the prediction results. For this baseline, we used a majority classifier, which predicts the majority (i.e. most frequent) class for each record. All code developed for this project is available at github (https://git.gesis.org/lisa.posch/geo-metadata-prediction).

## Results

This section describes the latent topics identified by LDA, as well as the results of the supervised classification experiments.

### GEO metadata topics

**Figure 4.**
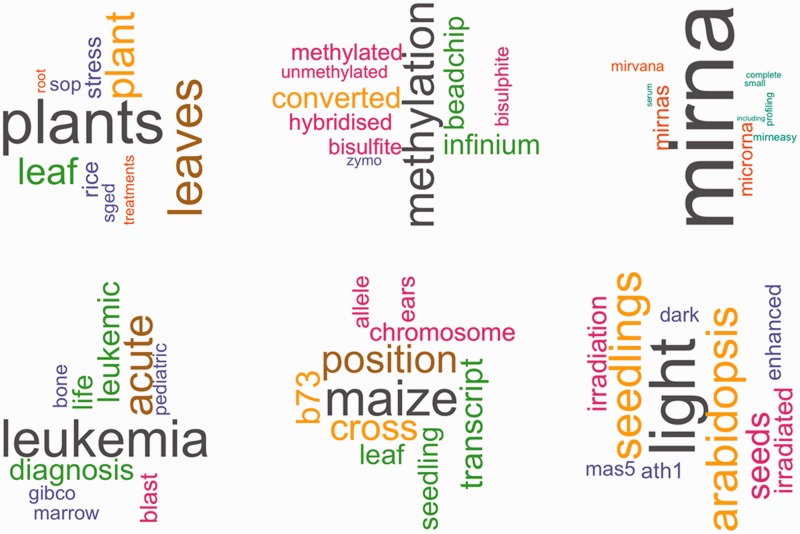
Top words for six sample topics. This figure shows the top 10 words for six of the 150 topics that were learned by LDA. Color and size of the words correspond to the relative importance of a word in the topic.

In LDA, a latent topic is defined by a probability distribution over the vocabulary. Words in a topic can therefore be ranked by the probability which they have in this topic, in order to visualize a topic by showing its most representative terms. Figure 4 depicts six topics from the 150 topics, showing the top 10 words for each topic (refer Supplementary data for a description of all topics). The size of the words corresponds to the importance of the word in the topic.

### Metadata prediction

**Figure 5.**
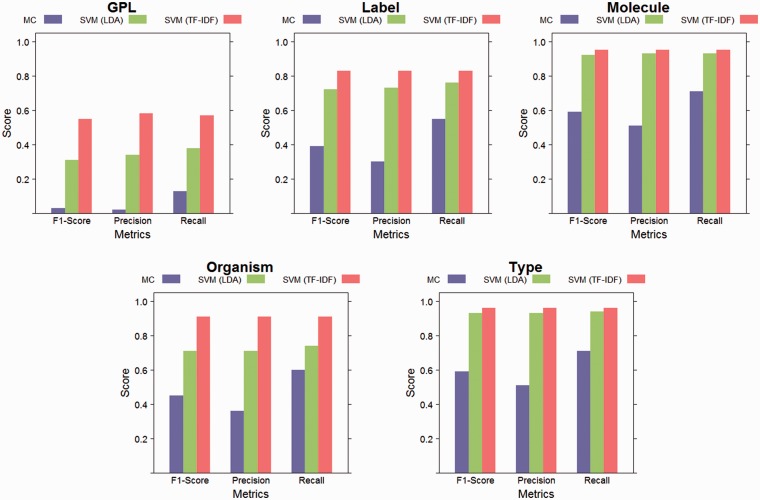
Evaluation results. This figure shows weighted class averages for precision, recall and F1-Score for each structured element. Results are reported for linear SVM with LDA features, linear SVM with TF-IDF features and for the majority classifier (MC) baseline. While some information was lost during LDA’s dimensionality reduction (by 98%), both approaches performed better than the baseline for all elements.

The performance of the SVM with linear kernel classifier, for both feature sets (latent topics and TF-IDF), as well as the performance of the majority classifier (MC) baseline are depicted in Figure 5. The best classification results were achieved for the elements with the smallest number of classes. The element with the highest number of classes, GPL, had the lowest absolute scores, but a large improvement over the baseline. During the dimensionality reduction of 98% by LDA, information is lost compared to using the full vocabulary as features for training the classifiers. However, the performance on elements with few values is similar as that of the classifiers trained on the full vocabulary. The baseline classifier is outperformed in all cases, both by the classifiers using TF-IDF features and the classifiers using topic features.

## Discussion

The results of our experiments indicate that unstructured metadata contains useful information for predicting structured metadata. This approach, which is not customized to the domain or specific dataset, is significant because elaborate descriptions are widely used in biomedical resources, while structured metadata is often incomplete, missing, inconsistent and even incoherent. Our results show that both TF-IDF and LDA models generated from textual descriptions in combination with supervised classification are an effective strategy to predict values for some structured metadata elements. TF-IDF on the full vocabulary resulted in the best performance. However this representation has the disadvantage of resulting in a very large dataset (over 70 000 features per document) that is not well semantically interpretable. Overall, our results offer a promising approach for metadata prediction that is likely to be applicable to other datasets, but more work is needed to assess its utility for different datasets.

Our classification setup achieved a better predictive performance than the baseline for all elements, despite the baseline, which predicts the majority class, being relatively high due to the heavily skewed class distribution of most elements. The goal of this work was not only to find the best supervised classifier for this task, but also to explore whether structured metadata elements can be predicted using the information from unstructured elements and whether topic distributions can successfully be used for metadata prediction in this domain.

One limitation of our experiments is that we did not use all values of the structured elements, but decided to exclude values which were used very infrequently. Future work is needed to investigate whether infrequently used values (<0.1% of the dataset) can be accurately predicted as well. In the experiments presented in this article, a fixed number of topics as well as fixed hyper-parameters were used, as LDA requires these parameters to be specified. An optimal number of topics and optimal hyper-parameters will likely lead to better prediction results. Therefore, future work is needed to further optimize these parameters. One planned approach is to use a non-parametric topic model using practical collapsed stochastic variational inference (8), as a memory friendly inference strategy that optimizes the number of topics as well as the hyper-parameters. Furthermore, LDA has been adapted and extended in many ways (9, 10). For example, a supervised form of LDA could be used which additionally takes into account the global popularity of the structured element values, in the form of a prior for the model.

Our work is highly relevant to both prospective and retrospective augmentation of metadata. In particular, the approach we describe here suggests that metadata authors and biocurators could be aided with accurate suggestions thereby reducing the time and effort to create rich, high quality metadata and help them scale to the increasing amount of submitted biomedical data. Furthermore, the predictions may help with correcting existing, wrongly entered element values: detected discrepancies between the element value entered by a user and the predicted value could be given to human curators for review. In line with the goals of the Center for Data Annotation and Retrieval (CEDAR) (11), we aim to incorporate this framework to improve the authoring of metadata. CEDAR is partnered with a number of community groups to further develop and evaluate our technologies including ImmPort, the data warehouse of immunology-related datasets led by Stanford (12), The Human Immunology Project Consortium (13), which channels experimental datasets to the ImmPort repository, The BioSharing Initiative (14) and The Stanford Digital Repository (15), operated by Stanford University Libraries, which helps all Stanford investigators to archive and disseminate data of all kinds. We plan to extend our context-sensitive recommendation engine (16) with the results of this work in order to provide improved real time support to metadata authors, particularly those that are following curation guidelines (17). Using a constellation of different technologies, such as what is proposed here, we will increase the accuracy of metadata values during submission, decrease the missing value problem and increase the consistency of metadata.

## Conclusions

We have developed a framework for the prediction of structured metadata, using unstructured text. We use TF-IDF and a topic model approach on the unstructured text contained in metadata records. These TF-DF values and the latent topics are then used as features for training supervised classifiers. The results of our experiments indicate that unstructured metadata elements contain information which can be successfully exploited for predicting structured metadata elements. Furthermore, our results suggest that topic models can infer meaningful topics from the unstructured text of metadata records, and that these topics can be successfully used as features for training supervised classifiers. While some discriminatory information is lost compared to using the full vocabulary as features for training the classifiers, the performance on elements with few values is close to that of the classifiers trained on the full vocabulary. Our work has implications for researchers and practitioners interested in biomedical metadata curation and metadata prediction.

## Supplementary data

Supplementary data are available at *Database* Online.

Supplementary Data
