# Predicting structured metadata from unstructured metadata

**DOI:** 10.1093/database/baw097

**Published:** 2016-06-13

**Authors:** Lisa Posch, Maryam Panahiazar, Michel Dumontier, Olivier Gevaert

*Database* 2016 (2016), doi:10.1093/database/baw080

The URL present in the abstract of the above article was included by mistake, and this has now been corrected online. The publisher apologizes for this error.

