# Supplementary material for: Predicting structured metadata from unstructured metadata
Source: Database (Oxford). 2016 May 17;2016:baw080. doi: 10.1093/database/baw080 (PMC4892825; doi:10.1093/database/baw080)
Supplement: Supplementary Data [file supp_2016_baw080_index.html]

Supplementary Data 

# Predicting structured metadata from unstructured metadata

## Supplementary Data

files

- Supplementary Data - txt file
